# Characteristics of the enzyme-induced release of bitter peptides from wheat gluten hydrolysates

**DOI:** 10.3389/fnut.2022.1022257

**Published:** 2022-10-04

**Authors:** Xiaorui Sun, Jiayi Zheng, Boye Liu, Zehua Huang, Fusheng Chen

**Affiliations:** ^1^College of Food Science and Engineering, Henan University of Technology, Zhengzhou, China; ^2^School of International Education, Henan University of Technology, Zhengzhou, China

**Keywords:** wheat gluten, bitter peptides, sensory evaluation, HPLC-MS/MS, peptide sequence

## Abstract

Bitter peptides in the enzymatic hydrolysates were prepared and purified from wheat gluten using aqueous ethanol solutions and macroporous resin, which has opened a new road for the extraction and separation of bitter peptides. This report contains the release regularity of bitter peptides and the factors affecting the change of bitter intensity during enzymatic hydrolysis, providing a scientific basis for the research on debitterizing method. In this study, the effects of different degrees of hydrolysis (DH) and enzyme active sites on the bitter peptide content and bitter taste thresholds were discussed. The relationship between amino acid composition, molecular weight distribution, surface hydrophobicity and bitter taste thresholds was extensively researched. The results showed the exposure of hydrophobic amino acids and the bitterness intensity of the hydrolysates increased as the DH increased, and the bitterness of wheat gluten hydrolysates (WGHs) hydrolyzed by Alcalase was stronger than that of Trypsin. According to correlation analysis, the proportion of total hydrophobic amino acid is the first factor that affects the sensory properties of bitter peptide, and the release content of bitter peptides and the content of total bitter amino acids are the second, following by the content of peptide in the molecular weight range of 500–1,000 Da and the surface hydrophobicity. The amino acid sequence of bitter peptides from WGHs were identified and predicted using high performance liquid chromatography-mass spectrometry (HPLC-MS/MS) and bioinformatics. It was found that the molecular weight of most of the peptides was below 1,500 Da, and the Q value was higher than 5.86 kJ/mol.

## Introduction

Wheat gluten (WG) is a relatively safe and inexpensive edible source of plant protein (1). The WGHs have broad application prospects in food industry (2, 3). Recent scientific evidence (4, 5) indicates that WGHs contain bioactive peptides that have potential benefits for human health. It is reported that WGHs can play physiological activities in the body such as antihypertensive effect (6, 7), oxidation resistance (8), analgesic effect (9) and so on. However, owing to the high proportion of hydrophobic amino acids in WG, the WGHs produced by enzymatic hydrolysis elicit bitter taste, which limits the acceptance of consumers and the range of applications in functional foods (10, 11).

In recent years, researches (12, 13) on eliminating the bitterness of enzymatic hydrolysates to develop more functional foods with high nutritional value have been reported successively (14). Enzymatic debittering is to cut off the hydrophobic amino acid at the end of the peptide by exo-peptidase (carboxypeptidase or aminopeptidase) to generate a free amino acid with low bitterness, and it is widely used for debittering of hydrolysates due to mild conditions and no loss of physiologically active components (15). Tong (16) used Protex 6L followed by Protease A 2SD in a two-step hydrolysis-assisted water extraction method to produce soybean protein hydrolysates with reduced bitterness and high nutritional value. However, there are few reports about bitter peptides and the bitter sensory properties of WGHs. The specific release mechanism of bitter peptides during enzymatic hydrolysis and the main factors affecting the bitterness are not clear (17). In order to expand the research of enzymatic debittering, it is of great significance to study the mechanism of bitterness release and the internal causes of bitterness changes during enzymatic hydrolysis of WG.

Previous research indicated that the bitter taste of peptides was caused by the hydrophobic groups interact with taste bud cells. The Q rule (18) proposed by Ney found that the bitterness of peptide was closely related to their hydrophobicity. According to Q rule, when the Q value of peptide is more than 5.86 kJ/mol, it will appear bitter. When the Q value is <5.44 kJ/mol, it will not appear bitter. Between the two levels, whether there is bitterness depends on the specific situation. In general, the bitterness intensity of peptides increases with the increase of exposure to hydrophobic amino acids (19). Besides, it is found that the amino acid composition, molecular weight distribution, peptide sequence and spatial structure of bitter peptide are important factors affecting the bitterness intensity (20). In order to explore the release characteristics of bitter peptides from WG, it is recommended to extract the bitter peptides from the hydrolysates for further research.

Based on the fact that bitter peptides are mostly composed of substances with strong hydrophobicity, the methods for separating bitter peptides from the enzymatic hydrolysates mainly include organic solvent extraction, macroporous resin adsorption, ultrafiltration, gel permeation chromatography, etc. (21). Organic solvent extraction can rapidly and simply extract bitter peptides based on the principle of hydrophobic bitter substances have high solubility in organic solvents, common solvents include isobutanol, ethanol and other organic solvents. Zhang (22) extracted bitter substances from corn puffs with 75% ethanol-25% water solution, the bitterness of the ethanol aqueous fraction was significantly higher than that of the insoluble residual. Therefore, ethanol is an ideal solvent for the extraction of bitter peptides, different concentrations of ethanol solution are used to separate the bitter components in WGHs (23). In order to further obtain high-purity bitter peptides, it is often necessary to separate and purify the extracted bitter peptides through a complex process. DA201-C macroporous adsorption resin is a non-polar resin that can separate and purify small molecular peptides with high recovery, high efficiency and low cost (24), which is suitable for further enrichment and purification of hydrophobic peptides extracted by ethanol. Cheison (25) purified whey protein hydrolysate with DA201-C non-polar macroporous adsorption resin and found it can effectively enrich bitter peptides from enzymatic hydrolysates.

In this study, WGHs with different DH hydrolyzed by Alcalase and Trypsin were prepared. The intention was to determine the effect of different DH and enzyme active sites of endopeptidase on the bitterness intensity of the resulting hydrolysates. A method that combining ethanol aqueous solution with macroporous adsorption resin was employed to extract bitter peptides from enzymatic hydrolysates, the effect of the amino acid composition, molecular weight distribution and average hydrophobicity of bitter peptides on bitter taste thresholds were studied. The main objective of the current research was to elucidate the changing characteristic of the bitterness intensity and clarify the internal reasons for the difference in bitterness of WGHs. Then sephadex gel (26) was used to separate bitter peptides according to different molecular weight ranges, the sequence of the components with the strongest bitterness were identified by HPLC-MS/MS with Byonic software (27). The number of peptide chains, length of peptide segments, average hydrophobicity and amino acid of peptide terminal of bitter peptides in different enzymatic hydrolysis schemes were analyzed, which can more clearly and intuitively explain the structural characteristics of bitter peptides in the hydrolysis system. The identification of the bitter peptides will provide a more targeted basis to develop bitterness reduction strategies in related enzymatic hydrolysates. This research on the mechanism of bitterness formation and release is of great significance to explore more advanced enzymatic hydrolysis technology, bitterness inhibition technology and quality control method of WG peptide suitable for industrial production.

## Experimental

### Materials and reagents

WG (79% protein) was purchased from Beijing Rui Mai Jia He Co., Ltd. (Beijing, China). Amylase, Alcalase 2.4 L and Trypsin were purchased from Novozymes (Beijing, China). All other chemicals and solvents were of analytical grade.

### Analysis methods

#### Preparation of wheat gluten hydrolysates

WG (15 g) was dissolved in 300 mL deionized water at thermostatic enzyme reactor, the suspension was adjusted to the appropriate temperature and pH depending on the respective enzyme used. Then, Alcalase (E/S = 1:20) and Trypsin (E/S = 1:20) was added to hydrolyze WG. During the hydrolysis process, the pH of the solution was kept constant by adding 1 mol/L sodium hydroxide. The proteolysis reaction was stopped at specific DH, with the hydrolysate immediately heat treatment in boiling water for 15 min. Then the resulting hydrolysates were centrifuged at 5,000 r/min for 15 min, the supernatants were freeze-dried and stored at 20°C (28). Alc-4, Alc-12 and Alc-20 are referred to as the enzymatic hydrolysates hydrolyzed by Alcalsse to 4%, 12% and 20%; Try-4, Try-12 and Try20, similarly.

#### Determination of degree of hydrolysis

The pH stat method was used to determine the DH of WGHs. The DH represents the percentage of broken peptide bonds (h) in the total peptide bonds (h_tot_) during protein hydrolysis (29). Owing to the release and absorption of protons in the process of protein hydrolysis, a certain amount of alkali must be added continuously to maintain the pH of the solution system. The amount of sodium hydroxide added can directly represent the degree of protein hydrolysis. Therefore, this method can control the amount of sodium hydroxide added to make the enzymatic hydrolysis reach the specified DH. The calculation formula is as follows:


$$\begin{array}{l} DH=B\times N_{b}\times \frac{1}{\alpha }\times \frac{1}{M_{p}}\times \frac{1}{h_{tot}}\times 100\% \end{array}$$

In the formula, B is the volume of alkali solution consumed in the hydrolysis process (mL); Nb is the molar concentration of sodium hydroxide (mol/L); Mp is the mass of protein in the substrate (g); H_tot_ is the total number of peptide bonds in protein (mmol/g); For WG, h_tot_ = 8.38 mmol/ g; α is the dissociation degree of the amino group as a function of reaction temperature.

#### Extraction of bitter peptides by ethanol fractional precipitation

Lyophilized WGH was dissolved in deionized water at the concentration of 20% (w/v). Anhydrous ethanol was slowly added to the enzymatic hydrolysate until the concentration of ethanol in the solution system reached 40%. Then the solution was thorough mixed and sediment was removed after centrifugation (5,000 r/min; 20 min). The supernatants were repeatedly extracted through 60 and 80% ethanol solution in accordance with the above operations. Finally, the ethanol extraction solvent was rotated and evaporated and the bitter peptide lyophilized powder was prepared. All the freeze-dried fractions were stored at 20°C before use (30).

#### Isolation and purification of bitter peptide

The extract was dissolved in ultra pure water at a concentration of 30 mg/mL and 30 mL was loaded onto DA201-C macroporous resin, the volume of the chromatographic column is 300 mL. The water-soluble impurities were eluted with ultra pure water, and then eluted with 80% volume fraction ethanol, which could completely elute the bitter peptides adsorbed on the column at a flow rate of 1 mL/min. The eluted components were collected at 220 nm and the ethanol solvent was removed by rotary evaporation, lyophilized to next step of separation. Alc-4M, Alc-12M, and Alc-20M are the bitter peptides purified from Alc-4, Alc-12 and Alc-20 by macroporous resin; Try-4M, Try-12M, and Try20M are the bitter peptides purified from Try-4, Try-12, and Try-20 by macroporous resin.

#### Sensory evaluation

Eight experienced sensory evaluators (four males and four females, ages 20–35) evaluated the taste attributes of WGHs and bitter peptides. The sample solution at a concentration of 1% was randomly presented to the trained sensory evaluators. The evaluator rated the bitterness intensity of samples on a 10-point line scale, anchored with caffeine references: 0.3, 0.8, and 1.2 g/L, corresponding to the bitterness intensity of 2, 5, and 10, respectively (22). The samples were scored according to the standard score and the bitterness of each sample was the average value of the evaluation results of each sensory evaluator. To ensure the accuracy of taste, 10% concentrated lemon juice and water were used as palate cleansers before each evaluation. If the bitterness still exists, evaluators can eat unsalted crackers to eliminate the bitterness in the mouth.

#### Release content of bitter peptide

Release content of bitter peptide


$$\begin{array}{l} =(The\;total\;protein\;content\;of\;macroporous\;resin\;extraction\\ \;components)/(Total\;protein\;content\;of\;raw\;materials)\times 100\% \end{array}$$

The total protein content was determined by Kjeldahl method (GB 5009.5-2016).

#### Amino acid composition

The amino acid compositions in the bitter peptides were analyzed using amino acid analyzer. A certain amount of sample was hydrolyzed at 110°C for 24 h with 6 mol/L HCl in Hydrolysis tubes, Then the sample was transferred into a 50 mL volumetric ask and filtered. The filtrate was fully mixed and evaporated in a vacuum desiccator to remove hydrochloric acid, the residue was dissolved in 1 ml of water and evaporated again. Then the dried sample was dissolved in the sample diluent, ultrasonic for 1 min and filtered into the sample bottle for further analysis.

#### Molecular weight determination

The molecular weight (MW) distributions of bitter peptides were determined by high-performance gel-filtration chromatography with a TSK gel G2000 SWXL (300 mm × 7.8 mm) column and Waters 1525 liquid chromatograph system. The mobile phase used is acetonitrile/water/trifluoroacetic acid (10/90/0.1, v/v/v). The sample was monitored at wavelength of 220 nm and a column temperature of 30°C and a flow rate of 0.5 ml/min (31). The standards used in the molecular weight correction curve are: Cytochrome C (MW 12,500 Da), Bacilli enzyme (MW 1,450 Da), tetrapeptide GGYR (451 Da), and tripeptide GGG (189 Da). The standard curve equation was is y = – 0.253x+7.04 (R2 = 0.9911).

#### Average hydrophobicity

The fluorescence probe method (32) was used to detect the surface hydrophobicity of bitter peptides from WG. The sample was dissolved in phosphate buffer solution with the concentration of 0.01% (w/v). The protein concentration was assessed using the Coomassie brilliant blue method with bovine serum albumin as standard. The protein solution was serially diluted with phosphate buffer (pH 7) to obtain the solutions with protein concentrations ranging from 0.0005 to 0.5%. 8-Anilino-1-naphthalenesulfonic acid (ANS) solution (20 μL) was added to the sample solution with different concentrations and the relative fluore scence intensity of ANS protein was measured with a spectrofluorometer using 338 nm and 496 nm as the excitation and emission wavelengths, respectively. The initial slope (H_o_) of the relative fluorescence intensity against protein concentration, calculated by linear regression analysis, was used as an index of protein surface hydrophobicity.

#### HPLC-MS/MS analysis of gel filtration chromatography product

Bitter peptides were subjected to further separation separated by Sephadex G-25 gel chromatography column according to different molecular weight ranges. To clarify the key peptides accounting for the bitter taste sensation, the components with the strongest bitterness were selected through sensory evaluation and were analyzed by HPLC-MS/MS (33). The liquid chromatography column was C18 reversed-phase analysis column (150 um i.d. × 150 mm, packed with Acclaim PepMap RPLC C18, 1.9 um, 100 Å); Mobile phase A was a mixture of 99.9% water and 0.1% formic acid, and mobile phase B was a mixture of 80% acetonitrile and 0.1% formic acid. The gradient of liquid phase elution was 0–2 min, 4–8%B; 2–45 min, 8–28%B; 45–55 min, 28–40%B; 55–56 min, 40–95%B; 56–66 min, 95%B, eluting at a constant flow rate of 600 nL/min. The sequence of peptides in the samples was performed by processing the auto multiple MS (Auto MS/MS) spectra program. Data dependent scanning mode was chosen to conduct full scanning acquisition in the orbital well with a resolution of 70,000 (AGC3e6), the ionization source ESI, mass scanning range was 300–1,800 m/z; capillary voltage was 2.3 kV; drying gas temperature was 320°C. Data were analyzed using Byonic software.

## Results and discussion

### Bitterness intensity of wheat gluten hydrolysates

**Figure 1:**
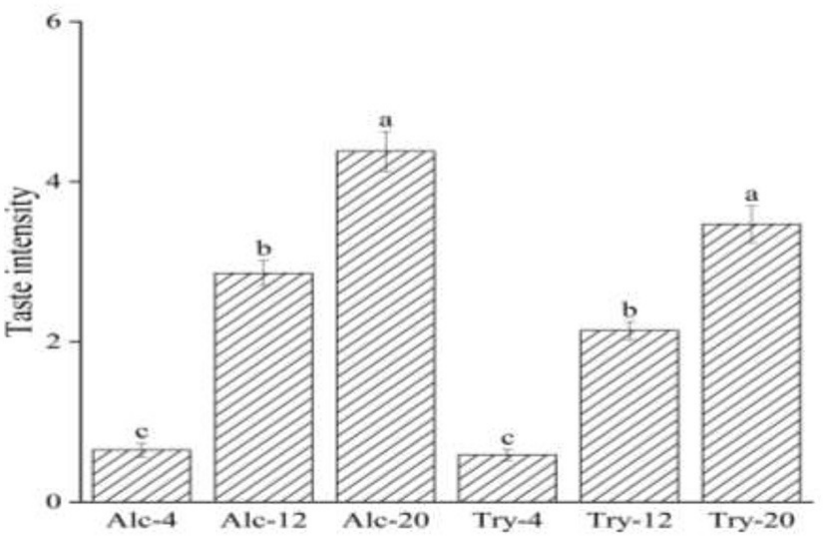
Bitterness intensity scores of wheat gluten hydrolysates at 4, 12, 20% degree of hydrolysis. The values in a column followed by different letters were significantly different (*p* < 0.05).

The bitterness intensity of enzymatic hydrolysates is closely related to the type of enzymes and the DH. Figure 1 presented the bitterness intensity of WGHs hydrolyzed by endopeptidases (Alcalase and Trypsin), which increased continuously as the DH increased. The hydrolysates had low bitterness at a low DH, such as bitterness intensity scores of Alc-4 and Try-4 were only 0.65 and 0.59, on the score of the hydrolysates were mostly macromolecular peptides with α-helix or irregular curl structure at initial stage of hydrolysis, the hydrophobic groups of these macromolecular peptides were embedded in the protein molecules and were not easy to contact with the taste receptors on the taste buds (34). During the process of continuous hydrolysis, the peptide chain was gradually opened and the peptide bond of macromolecular peptide was constantly broken, which facilitated the exposure of hydrophobic group and interacted with taste receptors to produce a strong bitterness sensing. When the DH reached a higher level, bitterness intensity scores of Alc-20 and Try-20 increased significantly to 4.38 and 3.4. Besides, the bitterness intensity of the hydrolysate is also affected by the specificity of protease, the bitterness intensity of hydrolysates prepared by different proteases was different at the same DH due to different enzyme active sites. For example, the bitterness of Alc-20 was higher than Try-20, this was because Alcalase was a hydrophobic and specific endopeptidase, and its enzyme active sites were aromatic amino acid residues such as tyrosine, tryptophan and phenylalanine, as well as the peptide bond at the carboxyl end of hydrophobic amino acid residues, resulting in a high proportion of hydrophobic amino acid at the peptide end. Researches are showing that the short peptide with hydrophobic amino acid at the end of the peptide chain have heavy bitterness (35). The enzyme active sites of Trypsin are lysine, arginine and other basic amino acids, which avoid the heavy bitterness caused by proline is in the middle of the peptide chain or adjacent to arginine (36).

### Release content and bitter taste thresholds of bitter peptides

**Figure 2:**
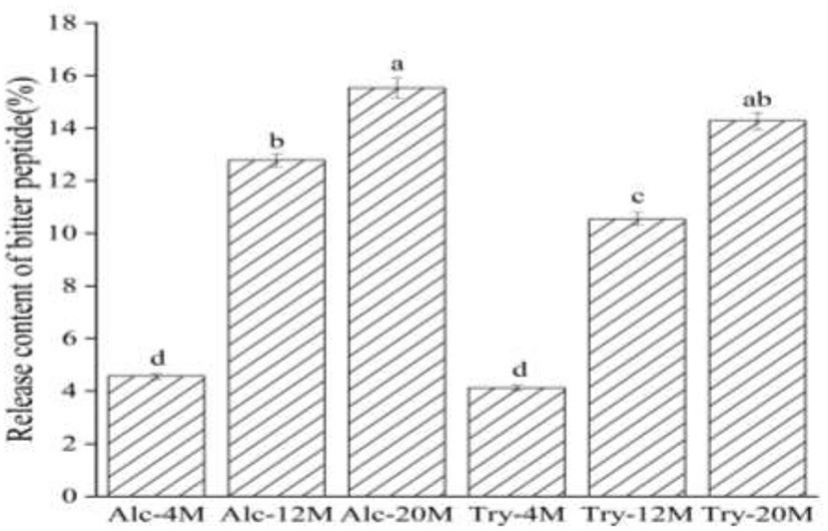
Release content of bitter peptides in different extracts. The values in a column followed by different letters were significantly different (*p* < 0.05).

The release content of bitter peptides during enzymatic hydrolysis of WG is the ratio of the total protein content of the macroporous resin extracted components to the total protein content of the raw materials. As shown in Figure 2, the release content of bitter peptides of Alc-4M and Try-4M was only about 4%, which suggested that the release content of bitter peptide was less at low DH. During the period of the DH from 4 to 12%, the release content of bitter peptides increased significantly. The release content of bitter peptides of Alc-12M and Try-12M was 12.78% and 10.55%, increased by about 6 and 8 percentage points, respectively. This is mainly due to the increase of the exposure of hydrophobic groups as a result of the increase of the DH, which is conducive to improving the proportion of hydrophobic amino acids in the peptide segment (37). Then the release content of bitter peptides of Alc-20 and Try-20 increased to 15.53 and 14.28%. The release content of bitter peptides from 4 to 12% DH increased faster than that from 12 to 20% DH, because the protease first acts on the protein with a relatively loose structure in the process of protein hydrolysis, the hydrolysis reaction speed is faster and the content of bitter peptides containing more hydrophobic amino acids increased rapidly at this time.

Although the increase of the DH promotes the release of more hydrophobic bitter peptides, it also helps to improve the peptide nitrogen recovery rate and raw material utilization rate. Therefore, the DH and protein recovery rate should be improved as much as possible on the premise of controlling the bitterness in the actual production. In addition, different enzyme activities and enzyme active sites also affect the content of bitter peptides. Alcalase is helpful to generate more hydrophobic bitter peptides because it can open the hydrophobic structure region of protein and expose more hydrophobic groups. It can be inferred that the bitterness of enzymatic hydrolysates is related to the release of bitter peptide from the same change trend.

**Figure 3:**
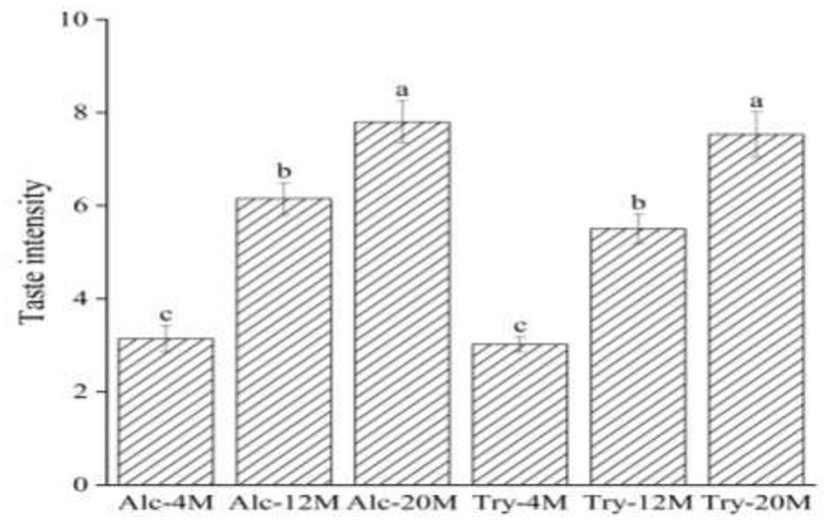
Bitterness intensity scores of different extracts. The values in a column followed by different letters were significantly different (*p* < 0.05).

The high bitterness intensity of bitter peptides indicates the small bitter taste thresholds of bitter peptides. Consequently, the bitterness intensity of the extracted components can reflect the bitter taste thresholds of bitter peptides. The bitter peptides with strong hydrophobicity from enzymatic hydrolysates were extracted by high concentration ethanol solution, and further purified by macroporous adsorption resin. As could be seen from Figure 3, the bitterness intensity of the extracts enriched by ethanol fractionation precipitation and macroporous resin was significantly higher than the bitterness intensity of corresponding original hydrolysates. The bitterness of Alc-20M and Try-20M were 7.80 and 7.53, increased by 43.85 and 54.85% compared with Alc-20 and Try-20. It was clear that ethanol sedimentation combined with macroporous adsorption resin was a simple but efficient method for extracting bitter peptides in the hydrolysates. And the overall change trend of bitterness intensity of extracted components was consistent with that of enzymatic hydrolysates, which suggested that the bitter taste thresholds of bitter peptides also determined the bitterness intensity of enzymatic hydrolysates. The amino acid composition, molecular weight distribution and hydrophobicity of these samples might be responsible for their taste, and the separation and purification of bitter peptides from the WGHs were helpful to analyze the factors affecting the bitter taste thresholds.

### Amino acid composition

Bitter peptide binding to bitter taste receptor must have two determining sites, namely binding unit and stimulating unit, and the space distance between the two sites should be maintained about 4.1 Å. The binding unit that determines the generation of bitterness contains at least three hydrophobic groups with large carbon chains, and the stimulating unit includes an α-amino group or hydrophobic group (38). Therefore, a high proportion of hydrophobic amino acids in the peptide increases the chance of interaction with taste receptors, which tends to cause the peptide to show a strong bitterness (16). In addition, Proline plays an important role in the presentation of bitterness. This is mainly because the imino ring structure of proline is easy to fold the peptide skeleton, the binding site and the stimulation site of the peptide are closer in space, and it is easier to interact with the bitter taste receptor and present a strong bitterness (39).

The amino acid composition of bitter peptides in hydrolysates hydrolyzed by Alcalase and Trypsin was shown in Table 1. Proline, Phenylalanine, Leucine, Valine, Isoleucine and other hydrophobic amino acids account for a high proportion of bitter peptides in different enzymatic hydrolysis schemes. From 4% DH to 20% DH, comparing the changes of total hydrophobic amino acids and total bitter amino acids in the bitter peptide, it was found that they both increased with the increase of DH. The contents of total hydrophobic amino acids and total bitter amino acids of Alc-4M were 38.03 and 42.31%, and those of Try-4M were 38.04% and 43.13%. When the DH reached 20%, the total hydrophobic amino acid content and total bitter amino acid content of Alc-20M increased by 6.96 and 7.84%, and those of Try-20M increased by 6.30 and 6.17%. This indicated that the regions with strong hydrophobic interaction gradually participated in the enzymatic hydrolysis reaction during the hydrolysis process, resulting in the increase of the contents of total hydrophobic amino acids and total bitter amino acids in bitter peptides. However, the proportion of total hydrophobic amino acids and total bitter amino acids in the bitter peptides prepared by Alcalase and Trypsin was no significant difference at the same DH. It showed that the specificity of enzyme active sites had little effect on the amino acid composition, and the difference in amino acid composition of the bitter peptides was mainly due to the different DH. According to the results of amino acid composition and bitterness of bitter peptides, it could be inferred that the content of hydrophobic amino acids and bitter amino acids in the peptide chain had an important impact on the bitter taste thresholds of the peptides. A high proportion of total hydrophobic amino acids in the peptide would decrease the bitter taste thresholds.

**Table 1:** Amino acid composition of macroporous adsorption resin extracts.

**Amino acid**	**Content percentage (%)**					
	**Alc-4M**	**Alc-12M**	**Alc-20M**	**Try-4M**	**Try-12M**	**Try-20M**
Asp	2.12 ± 0.09^a^	1.91 ± 0.12^a^	2.02 ± 0.09^a^	2.61 ± 0.08^a^	2.34 ± 0.11^a^	2.03 ± 0.13^a^
Thr	2.25 ± 0.03^ab^	2.23 ± 0.11^ab^	2.06 ± 0.11^b^	2.59 ± 0.03^a^	2.46 ± 0.03^a^	2.44 ± 0.03^a^
Ser	4.47 ± 0.21^a^	4.26 ± 0.23^a^	4.20 ± 0.23^a^	4.21 ± 0.21^a^	4.28 ± 0.22^a^	4.23 ± 0.22^a^
Glu	39.30 ± 1.15^a^	39.03 ± 1.25^a^	35.72 ± 1.25^b^	38.21 ± 1.15^a^	37.71 ± 1.07^a^	36.49 ± 1.15^a^
Gly	3.48 ± 0.12^a^	3.37 ± 0.12^a^	3.15 ± 0.12^ab^	3.09 ± 0.08^ab^	3.17 ± 0.08^ab^	2.76 ± 0.08^b^
Ala	2.43 ± 0.14^a^	2.38 ± 0.14^a^	2.22 ± 0.14^a^	2.31 ± 0.14^a^	2.36 ± 0.15^a^	2.06 ± 0.03^a^
Cys	0.55 ± 0.03^a^	0.46 ± 0.03^a^	0.47 ± 0.03^a^	0.55 ± 0.03^a^	0.60 ± 0.04^a^	0.69 ± 0.04^a^
Val	3.87 ± 0.03^b^	3.96 ± 0.03^b^	3.89 ± 0.03^b^	4.31 ± 0.03^a^	3.96 ± 0.03^b^	3.96 ± 0.03^b^
Met	1.40 ± 0.01^a^	1.54 ± 0.08^a^	1.52 ± 0.08^a^	1.39 ± 0.07^a^	1.48 ± 0.04^a^	1.25 ± 0.03^a^
Ile	3.83 ± 0.13^ab^	4.16 ± 0.14^a^	4.13 ± 0.14^a^	3.74 ± 0.13^ab^	4.16 ± 0.1^a^	4.33 ± 0.13^a^
Leu	6.70 ± 0.15^b^	6.53 ± 0.15^b^	7.26 ± 0.15^a^	6.63 ± 0.12^b^	7.08 ± 0.12^a^	7.44 ± 0.15^a^
Tyr	3.45 ± 0.16^b^	4.42 ± 0.16^a^	4.57 ± 0.16^a^	4.33 ± 0.15^a^	3.84 ± 0.13^b^	4.15 ± 0.13^a^
Phe	6.08 ± 0.19^b^	7.04 ± 0.19^ab^	7.02 ± 0.19^ab^	6.40 ± 0.17^b^	6.95 ± 0.16^ab^	7.51 ± 0.16^a^
His	2.99 ± 0.27^a^	2.67 ± 0.22a	2.77 ± 0.24^a^	2.72 ± 0.27^a^	2.81 ± 0.27^a^	2.74 ± 0.24^a^
Lys	0.72 ± 0.06^a^	0.40 ± 0.03^b^	0.83 ± 0.03^a^	0.92 ± 0.05^a^	0.81 ± 0.04^a^	0.80 ± 0.05^a^
Arg	2.32 ± 0.17^a^	1.40 ± 0.26^b^	2.36 ± 0.26^a^	2.52 ± 0.23^a^	2.31 ± 0.21^a^	2.09 ± 0.21^a^
Pro	14.05 ± 0.37^ab^	14.24 ± 0.33^ab^	15.81 ± 0.33^a^	13.47 ± 0.31^b^	13.69 ± 0.31^b^	15.02 ± 0.32^a^
Total hydrophobic amino acids	38.03 ± 1.14^b^	43.23 ± 1.18^a^	44.99 ± 1.18^a^	38.04 ± 1.05^b^	41.05 ± 0.99^ab^	44.34 ± 0.93^a^
Total bitter amino acid	42.31 ± 1.54^b^	46.37 ± 1.59^ab^	50.15 ± 1.61^a^	43.13 ± 1.53^b^	45.29 ± 1.41^ab^	49.30 ± 1.45^a^

Data are presented as the mean ± standard deviation, n = 3. Different superscripts within the same row indicate significant differences (p < 0.05).

Total of hydrophobic amino acids (HAAs): Gly, Ala, Val, Leu, Ile, Pro, Met, Phe.

Total bitter amino acids (BAAS): Val, Met, Ile, Leu, Tyr, Phe, His, Lys, Arg, Pro.

### Molecular weight distribution

The MW distribution of bitter peptides extracted from the hydrolysates of Alcalase and Trypsin was shown in Table 2. The fractions of the peptides with a MW ranging from 180 to 1,000 Da was the main component of bitter peptides. The proportion of macromolecular peptides with MW above 3,000 Da decreased gradually as the increase of the DH, those peptides had longer peptide chains and the hydrophobic groups were easily wrapped in the molecule, so it was difficult to enter taste bud cells to interact with bitterness receptors (40), the components with molecular weight <180 Da were mainly composed of free amino acids and also did not show bitter taste. The recently published research revealed that the bitterness of hydrolysates was caused by small peptides with strong hydrophobicity and peptides with a relative molecular weight of 500–1,000 Da had the strongest bitterness (41). This might be because the peptides with MW in the range of 500–1,000 Da generally contain 4–8 amino acids, binding sites and stimulation sites of bitter peptides were more likely to bind to bitter receptors and showed a strong bitter taste.

**Table 2:** The molecular weight distribution of peptides from macroporous adsorption resin extracts.

**Molecular weight range (Da)**	**Distribution (%)**					
	**Alc-4M**	**Alc-12M**	**Alc-20M**	**Tyr-4M**	**Tyr-12M**	**Tyr-20M**
≥10 000	1.72 ± 0.03^b^	0.75 ± 0.02^c^	0.32 ± 0.01^e^	1.85 ± 0.05^a^	0.53 ± 0.02^d^	0.32 ± 0.01^e^
6 000–10 000	8.41 ± 0.33^a^	1.14 ± 0.06^bc^	0.61 ± 0.01^c^	8.32 ± 0.29^c^	1.69 ± 0.07^b^	0.54 ± 0.02^c^
3 000–6 000	15.51 ± 1.02^a^	4.82 ± 0.27^c^	2.37 ± 0.11^d^	13.83 ± 0.38^b^	3.15 ± 0.12^cd^	3.16 ± 0.19^cd^
1 000–3 000	19.37 ± 1.14^a^	8.52 ± 0.34^b^	8.19 ± 0.29^b^	17.85 ± 1.51^a^	9.23 ± 0.42^b^	9.72 ± 0.47^b^
500–1 000	27.78 ± 1.86^b^	42.08 ± 0.97^a^	46.69 ± 1.67^a^	26.89 ± 1.85^b^	41.12 ± 1.03^a^	44.63 ± 2.38^a^
180–500	22.05 ± 2.18^b^	35.54 ± 2.39^a^	36.91 ± 2.17^a^	25.69 ± 2.05^b^	36.91 ± 1.58^a^	37.50 ± 1.73^a^
< 180	5.16 ± 0.36^bc^	7.15 ± 0.44^a^	4.91 ± 0.37^bc^	5.57 ± 0.39^b^	7.37 ± 0.41^a^	4.13 ± 0.32^c^

The values in the same row followed by different letters were significantly different (p < 0.05).

Compared to the bitter peptides with a low DH, Alc-20M and Tyr-20M have greatly improved the content of small peptides, the proportion of polypeptide with MW of 500–1,000 Da was 46.69 and 44.63%, which showed that the average relative MW of the peptides were rapidly reduced due to degradation by endopeptidases, the peptides with large MW were further hydrolyzed into peptides with smaller MW as the deepening of hydrolysis. The increase of the content of the oligopeptides with MW of 500–1,000 Da was consistent with the increase of the bitterness intensity of the corresponding bitter peptides, confirming the oligopeptides with MW of 500–1,000 Da resulted in a strong bitter taste. In addition, The MW distribution of bitter peptides hydrolyzed by Alcalase and Trypsin under the same DH was no significant difference, indicating that the difference of enzyme active sites had little effect on the MW distribution.

### Surface hydrophobicity

The surface hydrophobicity of peptides represents the binding degree between hydrophobic groups and polar solution environment, which can measure the number of hydrophobic amino acid residues exposed in the protein, and also show the balance between the hydrophilic/hydrophobic groups of the protein. The surface hydrophobic of extraction components was higher than that of the hydrolysates, because the conformation of the protein was changed in the extraction process of bitter peptides by ethanol and macroporous adsorption resin, then the hydrophobic groups exposed by the unfolding of protein structure were easy to combine with the non-polar solution environment, and the original hydrophilic groups outside the bitter peptide were relatively reduced (42).

**Figure 4:**
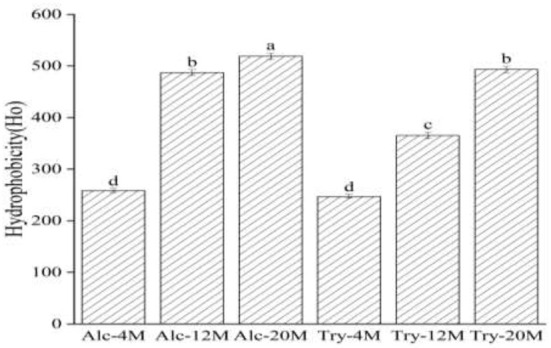
Surface hydrophobicity of macroporous adsorption resin extracts. The values in a column followed by different letters were significantly different (*p* < 0.05).

As could be seen from Figure 4, the surface hydrophobicity of Alc-4M and Tyr-4M were 258.39 and 247.09, the exposure of hydrophobic groups was not high under 4% DH on account of wheat protein was only partially hydrolyzed, and there was no significant difference between the bitter peptides prepared by Alcalase and trypsin. The surface hydrophobicity of bitter peptides increased with the increase of the DH. The surface hydrophobicity of Alc-20M and Tyr-20M increased to 518.73 and 493.12, especially the Alc-20M had more than doubled compared to Alc-4M. This was because with the deepening of hydrolysis, the protein structure continued to unfold and more hydrophobic amino acid residues were exposed. For example, Leucine, Proline and Phenylalanine were amino acids composed of alkanes or aromatic side chains, and their surface hydrophobic values were high. The surface hydrophobicity of Alc-20M and Tyr-20M also had significant differences, which might be due to the enzyme active sites of Alcalase were aromatic amino acid residues such as Tyr, Try, Phe and the carboxyl terminal peptide bond of hydrophobic amino acids, and the hydrolysis exposed more hydrophobic amino acid groups.

### Correlation analysis

**Figure 5:**
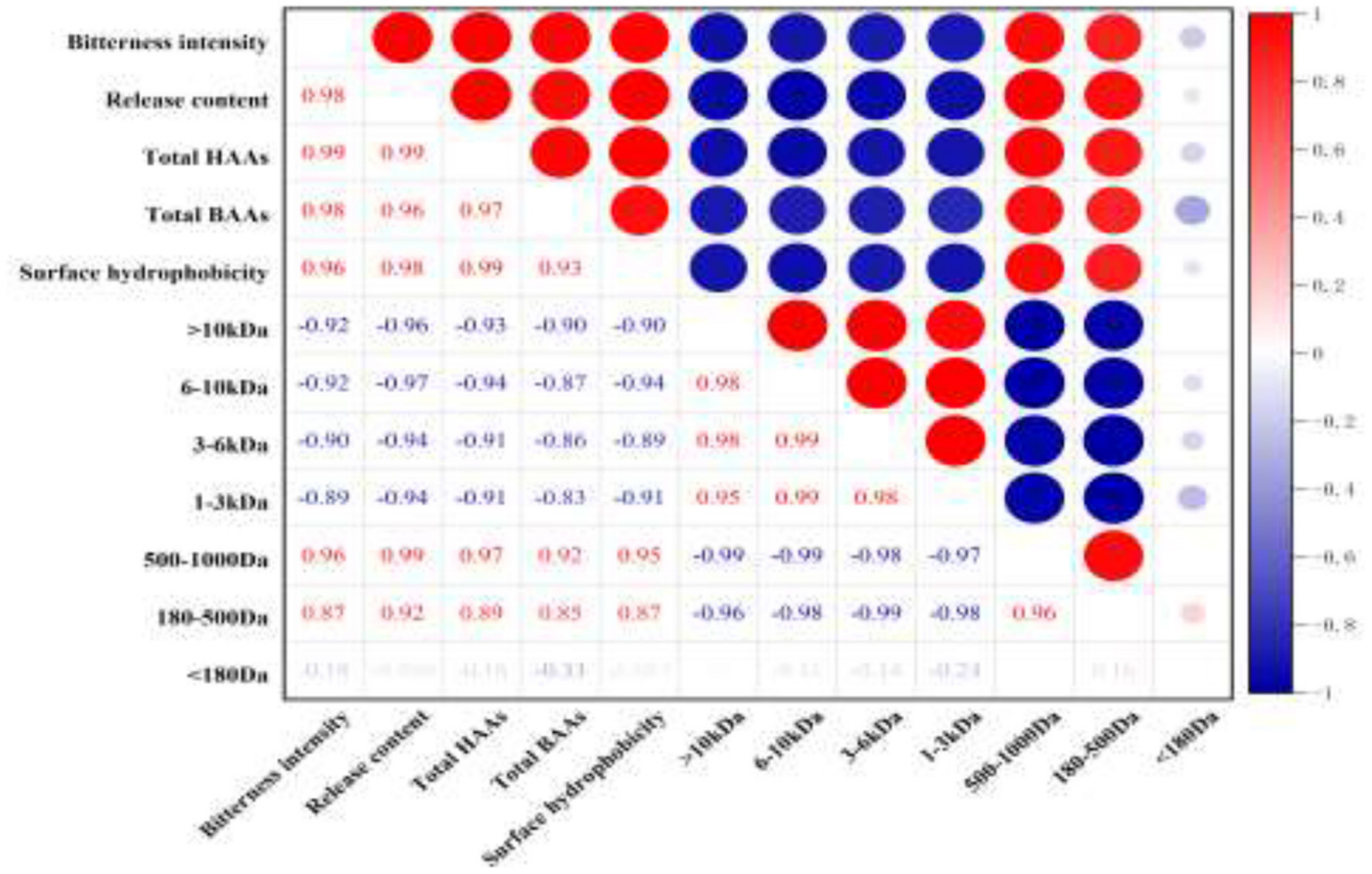
Heat map of correlation coefficient between different indicators (red represents positive correlation, blue represents negative correlation, and the depth of color reflects the size of correlation coefficient).

The correlation among bitterness intensity, the release content of bitter peptides, the proportion of total hydrophobic amino acid, the proportion of the total bitter amino acid, the percentage of peptides in different molecular weight ranges and surface hydrophobicity of peptides was analyzed. It could be seen from Figure 5 that the bitterness intensity of the WGHs was positively correlated with the release content of bitter peptides, the proportion of total hydrophobic amino acids, the proportion of total bitter amino acids, the surface hydrophobicity and the peptides with relative molecular weight between 500 and 1,000 Da (41).

The proportion of total hydrophobic amino acid in bitter peptide is the first index that affected the sensory properties of hydrolysates (*r* = 0.99). A high proportion of hydrophobic amino acids in a polypeptide will Make the bitterness heavier. The release content of bitter peptides and the content of total bitter amino acids are the second index that affected the sensory properties of bitter peptides (*r* = 0.98), followed by the peptides with molecular weight in the range of 500–1,000 Da and the surface hydrophobicity of peptides (*r* = 0.96). However, the peptides of molecular weight above 1,000 Da was negatively correlated with the bitterness of the hydrolysates, and the components with molecular weight <180 Da had a weak effect on the bitterness of hydrolysates. This indicated that the peptides with larger molecular weight and free amino acids had higher bitter taste thresholds, so they were not the factor causing the bitterness of the hydrolysates.

### Amino acid sequence of bitter peptides

The bitter peptides were further separated by Sephadex G-25 to reduce matrix interference and increase the feasibility and accuracy of sequence matching, then sequences of peptides were identified by HPLC-MS/MS and bioinformatics. The molecular weight, amino acid residues and average hydrophobicity of the peptides were shown in Tables 3–8, and the type and distribution of C-terminal and N-terminal amino acid residues were analyzed, which more intuitively and clearly illustrated the characteristics of bitter peptides. It was observed that identified bitter peptides were mainly small molecule hydrophobic peptides with high Q value, the amino acid composition of these peptides contains more hydrophobic amino acids, such as Tyrosine, Isoleucine, Tryptophan, Proline, Leucine, Valine and Phenylalanine. The bitter peptides from WGHs were all composed peptides with <16 amino acid residues, and the molecular weight of bitter peptides ranged from 477.26 to 1754.85 Da. The Q values of bitter peptides identified in Alcalase hydrolysates were >5.86 kJ/mol, and more than 77.78% of peptides identified in Trypsin hydrolysates were >5.86 kJ/mol.

**Table 3:** Result of peptides sequence of WGH-Alc4M determined by HPLC-MS/MS.

**No**.	**Amino acid sequence**	**Number of amino acid residues**	**Mw**	**Q value** **(KJ/mol)**
1	YPSYPQ	6	754.3406	7.61
2	LQPHQPF	7	866.4519	6.34
3	LQPFPS	6	688.3665	7.14
4	TITAPF	6	649.3556	6.87
5	FEEIRNL	7	920.4836	5.9
6	IPVIHPS	7	762.4509	8.01
7	NVYIPPY	7	865.4454	9.35

**Table 4:** Result of peptides sequence of WGH-Alc12M determined by HPLC-MS/MS.

**No**.	**Amino acid sequence**	**Number of amino acid residues**	**Mw**	**Q value (KJ/mol)**
1	YYPTSPQE	8	984.4309	6.23
2	LQPHQPF	7	866.4519	6.34
3	FLQPH	5	641.3406	6.77
4	QIPRQL	6	754.457	5.95
5	QLQPF	5	632.3402	6.27
6	IALPVPSQPVDPR	13	1388.79	6.82
7	IPVVQPS	7	739.4349	6.89
8	DVHVPPY	7	826.4094	7.49

**Table 5:** Result of peptides sequence of WGH-Alc20M determined by HPLC-MS/MS.

**No**.	**Amino acid sequence**	**Number of amino acid residues**	**Mw**	**Q value (KJ/mol)**
1	YPTSPQ	6	692.325	5.92
2	LPQLPYP	7	827.4662	9.25
3	LQPHQPF	7	866.4519	6.34
4	VPLY	4	491.2864	10.04
5	SIIQY	5	623.3399	7.32
6	FLQPH	5	641.3406	6.77
7	IFPQPQ	6	729.393	7.43
8	SPGKPYL	7	761.419	7.21

**Table 6:** Result of peptides sequence of WGH-Try4M determined by HPLC-MS/MS.

**No**.	**Amino acid sequence**	**Number of amino acid residues**	**Mw**	**Q value (KJ/mol)**
1	KVPIPNPS	8	851.4985	7.35
2	YLQADFK	7	884.4512	6.34
3	LPLQDVYK	8	975.551	7.30
4	VSIILPR	7	797.5244	8.03
5	VGHPEWEFPR	10	1253.606	6.23
6	ADIYNPR	7	848.4261	6.25
7	RQPGQGQPGYYPT	13	1448.692	4.66

**Table 7:** Result of peptides sequence of WGH-Try12M determined by HPLC-MS/MS.

**No**.	**Amino acid sequence**	**Number of amino acid residues**	**Mw**	**Q value (KJ/mol)**
1	KFDGILGL	8	862.5033	6.53
2	SFGQPQQQVPIEIR	13	1626.8598	4.91
3	PGVWEYV	7	849.4141	7.42
4	QLSQIPEQF	9	1089.5575	5.09
5	VGTMIEIPR	9	1015.5605	6.17
6	SLGLQLPF	8	874.5033	6.52
7	VVVDQFMLPK	10	1175.6493	6.69
8	KFDGILGL	8	862.5033	6.54
9	IEMPGPPY	8	903.4281	8.13
10	LPLQDVYK	8	975.551	7.30
11	PGVWEYV	7	849.4141	7.42
12	AMENEMLLR	9	1106.5333	4.65
13	PLFQLA	6	688.4028	7.49

**Table 8:** Result of peptides sequence of WGH-Try20M determined by HPLC-MS/MS.

**No**.	**Amino acid sequence**	**Number of amino acid residues**	**Mw**	**Q value (KJ/mol)**
1	QYEQTVVPPK	10	1188.626	5.77
2	RPQQPYPQPQ	10	1238.628	5.72
3	FEEIR	5	693.3566	6.23
4	LEVIR	5	629.3981	7.00
5	ADIYNPR	7	848.4261	6.25
6	VNVPLYR	7	860.4989	7.18
7	SIILPR	6	698.4559	8.19

**Figure 6:**
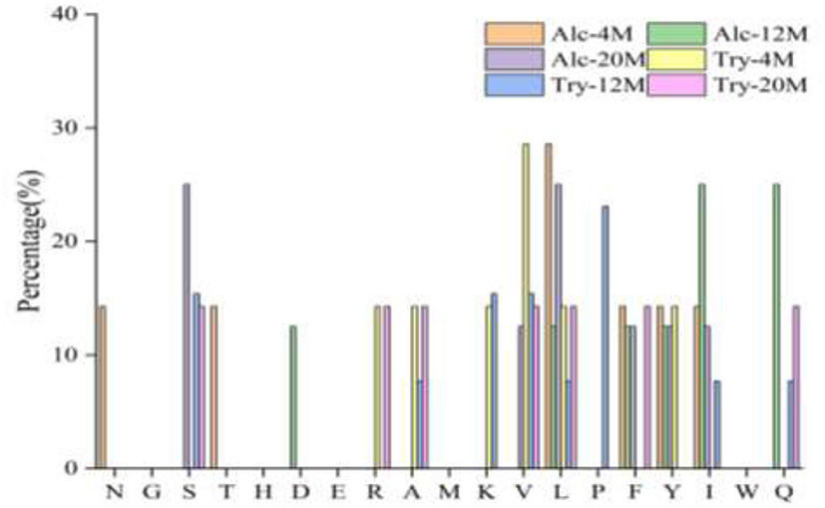
Statistic result of the kind and distribution of amino acid of C-terminal in the peptides.

**Figure 7:**
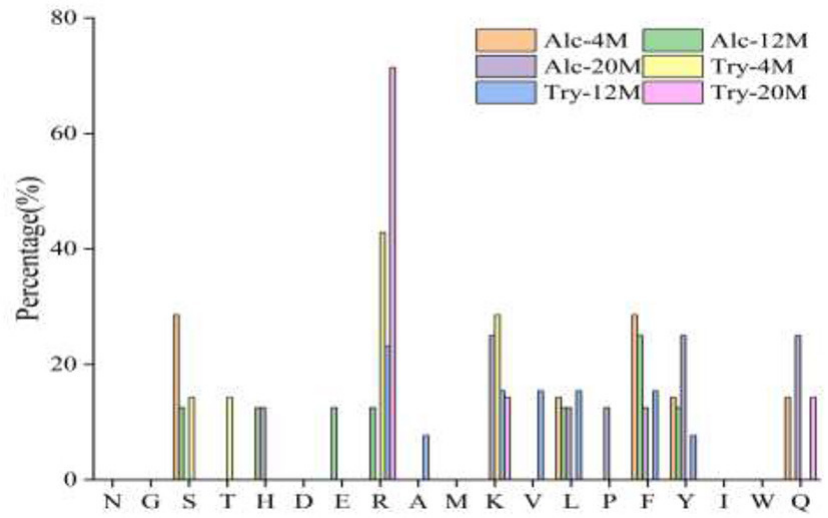
Statistic result of the kind and distribution of amino acid of N-terminal in the peptides.

Bitter peptides with hydrophobic amino acids, such as Ala, Phe, and Leu, increased the intensity of bitterness when they were located at the C-terminal position. It was reported that for short peptides, when hydrophobic amino acids were located at the C-terminal of the peptide chain and basic amino acids were located at the N-terminal of the peptides chains, the bitterness of the peptide was strong (43), and the bitterness of the bitter peptides would increase with the increase of the hydrophobic value of the C-terminal residue when the N-terminal of the peptides chains was a basic amino acid. It could be seen from Figures 6, 7 that hydrophobic amino acids (Trp, Phe, Ile, Try, Val, Leu, etc.) accounted for a high proportion in the C-terminal of the peptide and basic amino acids (Lys, Arg) account for a high proportion in the N-terminal of the bitter peptides. Comparing the terminal amino acid composition of bitter peptides hydrolyzed by Alcalase and Trypsin, it was found that the hydrophobic amino acids in the bitter peptides hydrolyzed by Alcalase have a high proportion in the C-terminal, and the basic amino acids in the bitter peptides hydrolyzed by Trypsin have a high proportion in the N-terminal, which was consistent with the known active site of the enzyme. Most of the amino acids at the end of the peptide chain were consistent with the known enzymes active sites. It is proved that Alcalase mainly cleaves the carboxyl terminal peptide bonds of aromatic amino acids and hydrophobic amino acids, and the enzymes active sites of Trypsin are basic amino acids.

## Conclusion

This study elucidated the release characteristics of bitter peptides during the enzymatic hydrolysis of WG, which provided a theoretical basis for the study of enzymatic debittering. Wheat gluten was hydrolyzed by Alcalase and Trypsin, the bitterness of the enzymatic hydrolysates was gradually increased with the increase of the DH, and the bitterness of the WHGs hydrolyzed by Alcalase was higher than that of Trypsin at the same DH. The bitterness intensity is affected by the release content of bitter peptides and the bitter taste thresholds. The release content of Alc-20M with the highest bitterness was 15.53%, and the bitterness intensity also increased to 7.8. The influencing factors of the bitter taste thresholds are sorted by the impact as the proportion of total hydrophobic amino acids in the peptides, the content of peptides with MW of 500–1,000 Da, and the surface hydrophobicity of the peptides. The total hydrophobic amino acids of Alc-20M was 44.99%, the content of oligopeptides with molecular weight between 500 and 1,000 Da was 46.69%, and the surface hydrophobicity was 518.73. The sequence of bitter peptides were identified by HPLC-MS/MS, the results showed that Alc-20M was composed of YPSYPQ, LQPHQPF, LQPFPS, TITAPF, FEEIRNL, IPVIHPS, NVYIPPY. It was found the detected sequences of the peptide are all composed of <15 amino acid residues. The relative molecular weight of bitter peptides was in the range of 491.29–1626.86 Da, and the average hydrophobicity of the peptides was basically above 5.86.

## Data availability statement

The original contributions presented in the study are included in the article/supplementary materials, further inquiries can be directed to the corresponding author/s.

## Author contributions

XS: conceptualization, software, visualization, investigation, writing—original draft, writing—reviewing and editing, and supervision. JZ: writing—original draft preparation. BL: conceptualization, funding acquisition, writing—reviewing and editing, and supervision. ZH: writing—original draft preparation and investigation. FC: conceptualization, investigation, writing—reviewing and editing, and supervision. All authors contributed to the article and approved the submitted version.

## Funding

This study was supported by the National Natural Science Foundation of China (Grant No. 31901640) and Cultivation Programme for Young Backbone Teachers in Henan University of Technology and Young Elite Scientists Sponsorship Program by Henan Association for Science and Technology (Grant No. 2021HYTP041).

## Conflict of interest

The authors declare that the research was conducted in the absence of any commercial or financial relationships that could be construed as a potential conflict of interest.

## Publisher's note

All claims expressed in this article are solely those of the authors and do not necessarily represent those of their affiliated organizations, or those of the publisher, the editors and the reviewers. Any product that may be evaluated in this article, or claim that may be made by its manufacturer, is not guaranteed or endorsed by the publisher.
